# Health-related quality of life in persons with West Nile virus infection: a longitudinal cohort study

**DOI:** 10.1186/s12955-017-0787-5

**Published:** 2017-10-23

**Authors:** Man Wah Yeung, George Tomlinson, Mark Loeb, Beate Sander

**Affiliations:** 1 Public Health Ontario, 480 University Avenue, Suite 300, Toronto, ON M5G 1V2 Canada; 2 Institute of Health Policy, Management and Evaluation, University of Toronto, 155 College St, Toronto, ON M5T 3M6 Canada; 3 Dalla Lana School of Public Health, University of Toronto, 155 College St, Toronto, ON M5T 3M6 Canada; 4 University Health Network, 200 Elizabeth Street, Toronto, ON M5G 2C4 Canada; 5 Department of Pathology and Molecular Medicine, McMaster University, 1280 Main Street West, Hamilton, ON L8S 4K1 Canada; 6 Institute for Clinical Evaluative Sciences, 2075 Bayview Ave, Toronto, ON M4N 3M5 Canada; 7 Toronto Health Economics and Technology Assessment (THETA) Collaborative, 200 Elizabeth Street, Toronto, ON M5G 2C4 Canada

**Keywords:** West Nile virus, Health-related quality of life, Short-form 36, Short-form 6D, Preferences

## Abstract

**Background:**

West Nile virus (WNV) infections are predominantly asymptomatic, although almost 1% become neuroinvasive and debilitating. We describe the impact of neuroinvasive and non-neuroinvasive disease on patient health-related quality of life (HRQoL).

**Methods:**

Short Form 36 questionnaire data came from a Canadian WNV cohort (Loeb 2008) of 154 patients followed for up to three years. We generated health utilities using the SF-6D. We calculated mean utility scores throughout follow-up and examined predictors using a linear mixed-effects model. We summarized HRQoL post-acute infection as: (i) long-term utility (mean of scores one year onward); (ii) area under the curve (AUC) one year onward. We examined predictors using beta regression. We used multiple imputation for sensitivity analysis.

**Results:**

Mean utility scores improved from 0.59 (95% CI: 0.38, 0.93) at baseline to 0.77 (0.53, 1) at six months, before plateauing for the remaining two years. Mean long-term utility was 0.81 (0.78, 0.85) and mean AUC was 0.80 (0.76, 0.84). Patients with neuroinvasive disease had consistently worse scores than their non-neuroinvasive counterparts, with the gap nearly closed after six months. After adjusting for confounding, neuroinvasive disease was not a significant predictor of HRQoL either throughout follow-up or post-acute infection. Rather, number of comorbidities and baseline utility scores were. Sensitivity analysis showed similar findings.

**Conclusions:**

Patients with WNV infection reported low HRQoL during acute illness, but improved rapidly by six months, regardless of neuroinvasive disease status. This is the first study reporting health utilities for WNV infection.

**Electronic supplementary material:**

The online version of this article (10.1186/s12955-017-0787-5) contains supplementary material, which is available to authorized users.

## Background

West Nile virus (WNV) has firmly established itself in North America since 1999. Symptomatic infections are mainly febrile (non-neuroinvasive), but almost 1% of infections become neuroinvasive [1], with debilitating physical and cognitive effects over the short and potentially long term.

There is a need to understand the impact of WNV infection from the patient perspective. Measures of health-related quality of life (HRQoL) capture the social, emotional and physical domains of the patient. HRQoL can be expressed as utility scores, which capture patient preferences for different health states and are central to calculating cost-utility in economic evaluations [2]. In WNV infection, few studies have examined physical and mental impairments and none have accounted for patient preferences [3, 4]. As such, we describe HRQoL as utility scores in patients with WNV infection from a cohort study, comparing those with neuroinvasive and non-neuroinvasive diseases. Further, we examined sociodemographic and clinical characteristics associated with HRQoL.

## Methods

### Ethics

The present work is part of a larger study, *Cost-effectiveness of West Nile virus intervention strategies. A computer simulation* (Canadian Institutes of Health Research grant: MOP133571), approved by the Ontario Agency for Health Protection and Promotion institutional review board. Data were obtained from a previous study from McMaster University (MOP-69010), which had received ethics approval from McMaster University, University of Manitoba, University of Saskatchewan, and University of Alberta [5].

### Data source

Data were obtained from an existing cohort study of 156 Canadian adults with confirmed WNV infection [5]. From provincial laboratory testing, patients with positive results were recruited by physicians and confirmed to have WNV through hospital, clinic, and laboratory records. Patients were predominantly from Ontario (65%), as well as Manitoba, Saskatchewan, and Alberta. Data were collected on sociodemographics, and comorbid diagnoses including cardiac disease, peripheral vascular disease, chronic obstructive, pulmonary disease, diabetes, renal failure, peptic ulcer disease, cancer, and rheumatologic disease. The study aimed to recruit patients within four weeks of symptom onset and interviewed patients during home visits or in ambulatory care over their follow-up of up to three years. The study measured patient HRQoL using the Medical Outcomes Survey Short Form 36 (SF-36) questionnaire, which consists of 36 questions that can be summed into eight subscales (physical functioning, social role functioning, bodily pain, general health perceptions, role limitations due to physical health, role limitation due to emotional problems, mental health, vitality) [6]. Each subscale has a score from 0 (maximum disability) to 100 (no disability). The eight subscales can be further aggregated into the Physical Component Summary and Mental Component Summary scores, which also range from 0 (maximum disability) to 100 (no disability).

### Measurements

#### Exposure

The diagnostic criteria for neuroinvasive diseases (encephalitis, meningitis, meningoencephalitis, and acute flaccid paralysis) are described elsewhere [5]. Patients who did not meet these criteria were classified having non-neuroinvasive disease.

#### Outcomes

The SF-36 questionnaire used is not based on preferences and therefore does not directly provide utility scores. To generate utility scores, we converted responses using the Medical Outcomes Study Short Form 6D classification (SF-6D) obtained from the University of Sheffield [7]. The algorithm combines selected SF-36 items into six health subscales (physical functioning, social functioning, bodily pain, role participation, mental health, vitality). The SF-6D uses preference weights from the United Kingdom general population based on the standard gamble technique, where individuals choose between a hypothetical outcome to occur with certainty (i.e., suboptimal health) or a gamble (i.e., probability of either perfect health or sudden death) [8]. Parametric and non-parametric models have been developed to predict all 18,000 health states described by the SF-6D [7, 9]. Utility scores range from 0 (equivalent to being dead) to 1 (equivalent to perfect health).

### Statistical analysis

We performed analyses in SAS version 9.3 (SAS Institute Inc., Cary, NC, USA) and R 3.3.2 [10]. Continuous variables were compared between those with and without neuroinvasive disease using Student’s t-test. Categorical variables were compared using the Chi-squared test or Fisher’s exact test. In the main analyses, we used all available data but excluded patients who did not complete the SF-36 questionnaire at any follow-up visit.

#### Mean utilities by time

We calculated the mean utility scores at different time points over their follow-up visits in strata formed by sex and baseline values of neuroinvasive disease status, age, and comorbidities. To calculate the 95% confidence intervals (CI), we used the 2.5th and 97.5th percentiles from 2000 bootstrap samples.

#### Predictors of HRQoL over entire follow-up

We modelled both logit-transformed utility scores (to account for the data being bounded by the open interval (0, 1)) and untransformed utility scores (for easier interpretability). To examine the association of patient characteristics with utility scores over time, we fitted linear mixed-effects models to all observations after baseline. All models had fixed effects for time (elapsed since baseline) and random effects for the intercept and time. Additional fixed effects considered were neuroinvasive disease status, age, sex, number of comorbid conditions, baseline utility scores, and the interaction of neuroinvasive disease with time. Each covariate was added alone to the base model and two additional models were fitted: one with all covariates, and one with all covariates except the interaction. Correlation between residuals was addressed through use of either an autoregressive (AR1) structure or an exponential power function based on time between measurements. As a further check on the results of the logit-transformed model, a beta-regression based random effects model was also fitted.

#### Response feature analysis

We collapsed a patient’s repeated HRQoL measures into single indices known as response features [11], focusing on two longer-term intervals: (i) from six months onward, or (ii) from one year onward The first response feature was the mean score over observations in the interval. The second response feature was the area under the curve (AUC), calculated using the trapezoid rule and then divided by the relevant follow-up time [11, 12]. This assumes that missing values fell linearly between existing observations. AUC can be interpreted as a time-weighted average HRQoL. These response features represented HRQoL post-acute infection, with larger values signifying better HRQoL.

#### Predictors of HRQoL response features

We used beta regression to examine the association between neuroinvasive disease and HRQoL post-acute infection. Beta regression accounts for the HRQoL data being bounded by the open unit interval (0, 1). HRQoL scores of one were adjusted to slightly smaller values (0.995) [13]. Specifically, we regressed mean long-term utility scores or AUC scores on neuroinvasive disease, adjusting for age (centered at 50 years old), sex, number of comorbid conditions, and baseline utility scores (centered at 0.5). Baseline utilities act as a proxy for other unmeasured confounders. We used the R betareg package [14] with a logit link, with and without parameterization of the precision as a function of the predictors [13].

### Sensitivity analysis

Utility score outcomes were missing when specific SF-36 responses were not completed or when patients missed study visits. Given attrition of the cohort, we checked for patterns of missing data across visits and determined whether having missing data at one visit was related to utility scores from the previous visit. We used multiple imputation by predictive mean matching, running 50 sets of imputation and taking the 100th sequential sample as the imputed dataset. We recalculated the response features on each imputed dataset, reran the beta regression model on each dataset and combined the estimated coefficients using Rubin’s rules [15].

### Role of the funding source

The funder had no role in the study design, analysis, decision to publish, or manuscript preparation. The corresponding author had full access to the data and had final responsibility for the decision to publish.

## Results

**Fig. 1:**
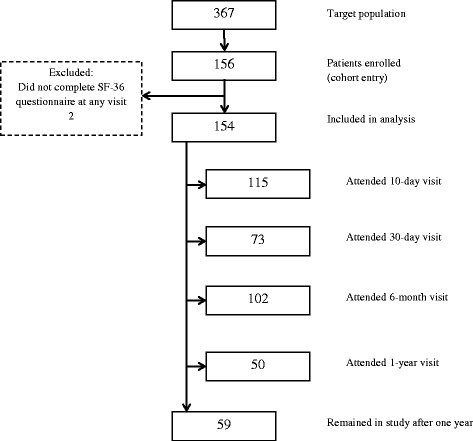
Study flow

The cohort enrolled 156 patients. Two patients who did not complete the SF-36 questionnaire at any visit were excluded for a final sample of 154 patients (Fig. 1) [5]. Patients were middle-aged with few comorbid conditions (mean: 0.9, standard deviation: 0.1) (Table 1). Sixty-two patients (40%) had neuroinvasive disease, and the remaining had non-neuroinvasive disease. There were 108 patients (70%) followed past six months, and 59 (38%) past one year. There were no discernible patterns in the missing HRQoL outcome across visits.

**Table 1:** Patient characteristics at cohort entry

	Total (*n* = 154)	Disease classification	
		Neuroinvasive (*n* = 62)	Nonneuroinvasive (*n* = 92)
Mean age (sd), years^a^	52 (13)	55 (15)	50 (12)
Female (%)	75 (49)	26 (42)	49 (53)
Mean follow up (sd), months^a^	11 (9)	11 (6)	9 (5)
Lost to follow-up at one year (%)	95 (62)	32 (52)	63 (68)
Comorbid conditions (%):			
Asthma	14 (9)	4 (6)	10 (11)
COPD^a^	4 (3)	4 (6)	0 (0)
Cancer	17 (11)	5 (8)	12 (13)
Cardiac disease	21 (14)	12 (19)	9 (10)
Cerebrovascular disease	3 (2)	2 (3)	1 (1)
Diabetes^a^	11 (7)	9 (15)	2 (2)
Liver disease^a^	6 (4)	5 (8)	1 (1)
Lung disease	16 (10)	9 (15)	7 (8)
Renal disease	7 (5)	4 (6)	3 (3)
Transplant	5 (3)	3 (5)	2 (2)
Mean number of comorbid conditions (sd)^a^	0.9 (0.10)	1.2 (0.2)	0.7 (0.1)
Prior hospitalizations (%)	118 (77)	51 (82)	67 (73)
Disease classification (%)			
Neuroinvasive	62 (40)	–	–
Encephalitis	21 (14)	–	–
Meningitis	3 (2)	–	–
Acute flaccid paralysis	6 (4)	–	–
Meningo-encephalitis	32 (21)	–	–
Nonneuroinvasive	92 (60)	–	–

Abbreviations: sd, standard deviation; COPD, chronic obstructive pulmonary disease

^a^Significant differences (*p* < 0.05) between patients with neuroinvasive and nonneuroinvasive disease. *P* value from independent samples t-tests

### Statistical analysis

#### Mean utilities by time

**Fig. 2:**
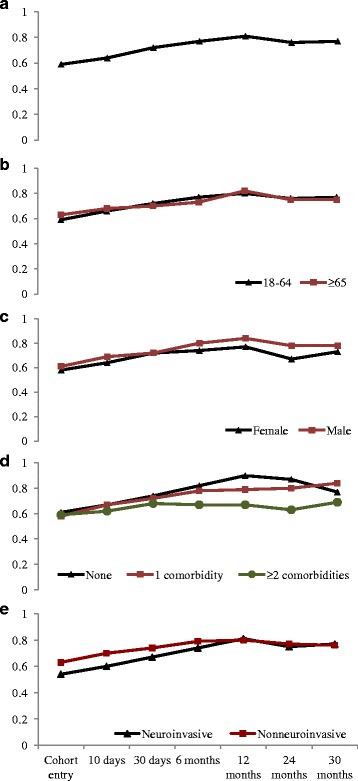
Mean utility scores over time for patients with at least one year of follow-up (Panel **a**) and for patients stratified by age (Panel **b**), sex (Panel **c**), number of comorbidities (Panel **d**), and neuroinvasive disease status (Panel **e**)

At baseline, the entire cohort had a mean utility score of 0.59 (95% CI: 0.57, 0.62), which improved rapidly over the subsequent months (Table 2). Qualitatively, the utility scores plateaued at six months at 0.77 (95% CI: 0.74, 0.80), and remained fairly steady over the remaining two years. The trend was similar in the subset of patients with at least one year of follow-up, and in patients stratified by age, sex, and comorbidity (Fig. 2). Specifically for patients with neuroinvasive disease, the mean utility score at baseline (0.54; 95% CI: 0.51, 0.57) was lower than that of their non-neuroinvasive counterparts (0.63; 95% CI: 0.60, 0.66). By one year however, their HRQoL improved to the levels of those with non-neuroinvasive disease (0.81 (95% CI: 0.76, 0.86) versus 0.80 (95% CI: 0.73, 0.85)).

**Table 2:** Mean utilities by time: Utility scores^a^ (95% confidence interval) at select study visits

	Cohort entry	10-day visit	30-day visit	6-month visit	12-month visit	24-month visit	30-month visit
Total cohort	0.59 (0.57, 0.62) *n* = 146	0.67 (0.64, 0.69) *n* = 106	0.72 (0.69, 0.75) *n* = 68	0.77 (0.74, 0.80) *n* = 95	0.80 (0.76, 0.84) *n* = 49	0.76 (0.66, 0.84) *n* = 12	0.77 (0.69, 0.84) *n* = 10
Neuro-invasive	0.54 (0.51, 0.57) *n* = 58	0.60 (0.57, 0.64) *n* = 38	0.67 (0.63, 0.72) *n* = 21	0.74 (0.69, 0.78) *n* = 39	0.81 (0.76, 0.86) *n* = 26	0.75 (0.65, 0.85) *n* = 8	0.77 (0.68, 0.86) *n* = 6
Non-neuro-invasive	0.63 (0.60, 0.66) *n* = 88	0.70 (0.67, 0.73) *n* = 68	0.74 (0.70, 0.78) *n* = 47	0.79 (0.76, 0.83) *n* = 56	0.80 (0.73, 0.85) *n* = 23	0.77 (0.57, 0.89) *n* = 4	0.76 (0.61, 0.86) *n* = 4

^a^Utility scores range on a scale from 0 (equivalent to being dead) to 1 (equivalent to perfect health). Larger values indicate better health-related quality of life. Scores are derived from the Medical Outcomes Study Short-Form-6D health state classification

#### Predictors of HRQoL over entire follow-up

Table 3 shows the linear mixed-effects model results for logit-transformed utility scores (Additional File 1 for untransformed scores). Results of the beta-regression based random effects model were similar (not shown). Neuroinvasive disease, gender, baseline utility scores, and number of comorbidities were significant predictors of HRQoL in the univariate analysis. However in the final model including all covariates, neuroinvasive disease status was no longer significant. It only appeared significant when baseline scores were excluded from the model.

**Table 3:** Linear mixed-effects model for predictors of health-related quality of life over entire follow-up

	Fixed effects (95% confidence interval)		
	Time only model	One additional covariate in model	All covariates in model
Intercept	1.07 (0.95, 1.20)	(varies)	1.00 (0.80, 1.20)
Neuroinvasive disease	–	**−0.36** **(−0.60, −0.11)**	−0.09 (−0.32, 0.13)
Age (per 10 years), centered at 50 years	–	−0.05 (−0.14, 0.04)	−0.01 (−0.09, 0.07)
Male	–	0.25 (0.0, 0.48)	**0.25** **(0.04, 0.45)**
Number of comorbid conditions	–	**−0.24** **(−0.34, −0.14)**	**−0.23** **(−0.32, −0.13)**
Baseline utility score, centered at 0.50	–	**2.41** **(1.6,0 3.21)**	**2.17** **(1.38, 2.95)**
Time elapsed since baseline (years)	0.73 (0.56, 0.90)	(varies)	0.59 (0.38, 0.80)
Interaction between neuroinvasive disease and years elapsed	–	–	0.02 (−0.28, 0.31)

Results are measured as logit-transformed utility scores. Utility scores range on a scale from 0 (equivalent to death) to 1 (equivalent to perfect health)

Significant results are bolded

The coefficients represent the change in the mean logit of utility scores when the predictor increases by one unit and the remaining covariates are held constant

In the final model, the intercept represents the mean logit HRQoL score when all covariates, and random effects are zero—that is, the logit HRQoL of a 50 year old female patient with non-neuroinvasive disease, no comorbidities, and a baseline utility score of 0.50. The intercept of 1.00 would be interpreted as a fitted utility score of $\frac{e^{1.00}}{1+{e}^{1.00}}$= 0.73. Different patient characteristics would vary one’s absolute HRQoL. For instance, a 60 year old male with non-neuroinvasive disease, one comorbid condition, and a baseline utility score of 0.60 would have the following logit score 1.00 (intercept) + 0 (non-neuroinvasive) – 0.01 (ten years greater than 50 years old) + 0.25 (male) – 0.23 (one comorbidity) + 2.17 * 0.10 (baseline utility was 0.10 greater than 0.50) = 1.247, corresponding to a fitted utility score of 0.78.

#### Response feature analysis

Patients in the total cohort had similar AUC (Table 4) and mean long-term utilities (Additional File 2). Utilities were also similar regardless of the time period captured. Time-weighted-AUC scores were 0.78 (95% CI: 0.75, 0.80) from six months onward, and 0.81 (95% CI: 0.78, 0.84) from one year onward. Patients with neuroinvasive disease had consistently lower HRQoL scores than those with non-neuroinvasive disease.

**Table 4:** Response feature analysis: Utility scores^a^ (95% confidence interval) summarized into area under the curve

	Area under the curve^b^	
	Six months onward	One year onward
Total cohort	0.78 (0.76, 0.81) *n* = 108	0.81 (0.77, 0.84) *n* = 59
Neuroinvasive	0.75 (0.71, 0.79) *n* = 45	0.80 (0.75, 0.85) *n* = 30
Nonneuroinvasive	0.80 (0.77, 0.83) *n* = 56	0.82 (0.76, 0.86) *n* = 29

^a^Utility scores range on a scale from 0 (equivalent to being dead) to 1 (equivalent to perfect health). Larger values indicate better health-related quality of life. Scores are derived from the Medical Outcomes Study Short-Form-6D health state classification

Note: One patient missing utility scores at all visits

^b^Area under the curve is the integral of the utility-time curve. Areas are time-weighted (Total area after one year ÷ follow-up time)

#### Predictors of HRQoL response features

**Fig. 3:**
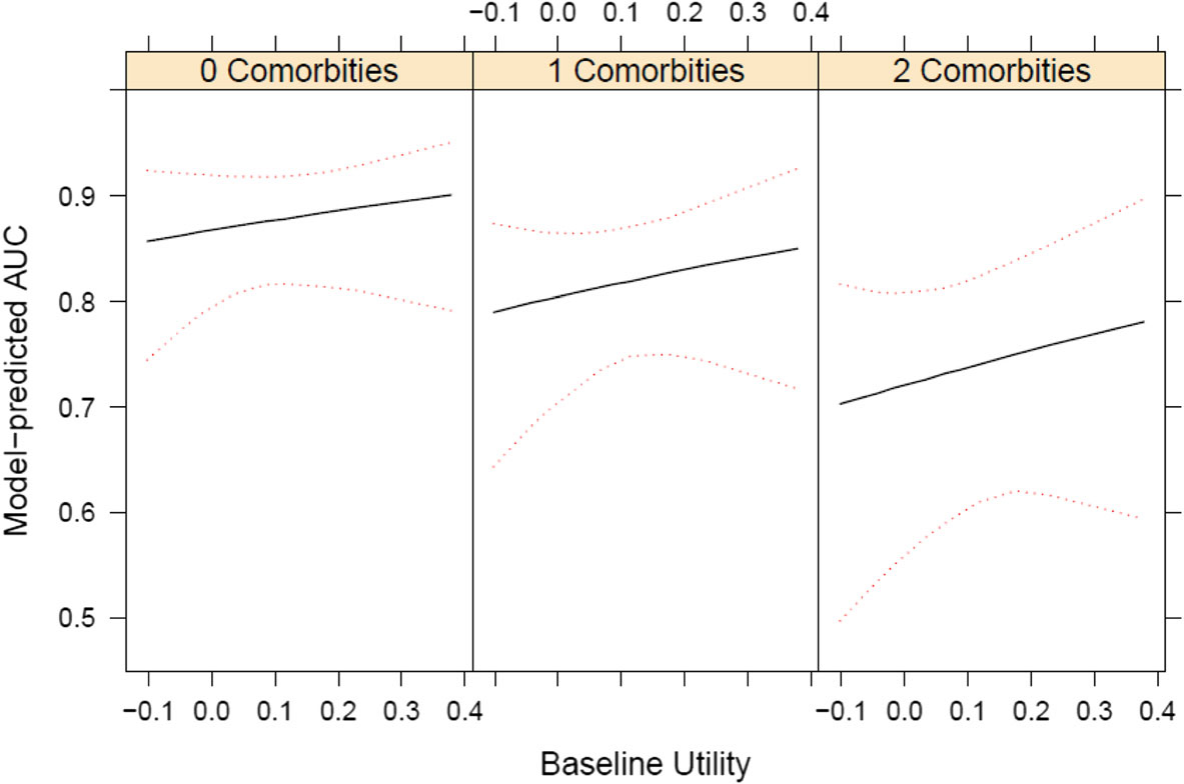
Predicted values from best beta-regression model for area under the curve (AUC) past one year with bootstrap confidence intervals

Table 5 shows the beta regression results for AUC, with and without multiple imputation. Number of comorbidities, and baseline utility scores were statistically significant for AUC from six months onward. However, only comorbidities were significant from one year onward. Effect sizes were reduced with imputation. Figure 3 shows the effect of each additional comorbid condition in a patient. The effect of comorbid conditions is as large as moving from the 5th to 95th percentile of observed baseline utilities. Neuroinvasive disease status did not have as much effect on predicted AUC as the number of comorbidities (Additional File 3).

**Table 5:** Beta regression model for predictors of health-related quality of life summarized into area under the curve^a^

	Coefficients^b^ (95% confidence interval)			
	No imputation		Multiple imputation	
	Six months onward	One year onward	Six months onward	One year onward
Intercept	1.26 (0.98, 1.54)	1.91 (1.66, 2.16)	1.31 (1.12, 1.49)	1.39 (1.19, 1.58)
Neuroinvasive disease	0.03 (−0.27, 0.34)	0.04 (−0.23, 0.30)	0.02 (−0.17, 0.22)	0.01 (−0.18, 0.19)
Age per 10 years, centered at 50 years	−0.08 (−0.19, 0.03)	−0.08 (−0.18, 0.014)	−0.03 (−0.10, 0.04)	−0.02 (−0.09, 0.05)
Male	0.26 (−0.02, 0.53)	0.02 (−0.29, 0.33)	0.15 (−0.03, 0.33)	0.10 (−0.08, 0.27)
Number of comorbid conditions	−0.25 (−0.35, −0.14)	−0.46 (−0.62, −0.30)	−0.16 (−0.24, −0.09)	−0.13 (−0.21, −0.05)
Baseline utility (centred)	1.70 (0.74, 2.67)	0.58 (−0.52, 1.68)	0.84 (0.06, 1.62)	0.29 (−0.61, 1.18)

Statistically significant results are in bold

^a^Area under the curve is the integral of the utility-time curve after one year. Areas are time-weighted (Total area after one year ÷ follow-up time)

^b^Coefficients and patient characteristics can be substituted into the following equation to calculate the area under the curve: $$ {\displaystyle \begin{array}{l} Logit\left( Mean\left( Utility score\right)\right)={\beta}_0+{\beta}_1\left(\mathrm{Neuroinvasive}\  \mathrm{disease}\right)+{\beta}_2\left(\frac{\mathrm{Age}-50\ \mathrm{years}\ \mathrm{old}}{10}\right)+{\beta}_3\left(\mathrm{male}\right)+\\ {}{\beta}_4\left(\mathrm{No}.\mathrm{comorbid}\  \mathrm{conditions}\right)+{\beta}_5\left(\mathrm{Baseline}\  \mathrm{utility}\  \mathrm{score}-0.50\right)\end{array}} $$For example, the regression equation for area under the curve past six months in a 60 year old male with neuroinvasive disease, one comorbid condition and a baseline utility score of 0.60 would be: 1.31 (intercept) + 0.02 (neuroinvasive) – 0.03 (ten years greater than 50 years old) + 0.15 (male) – 0.16 (one comorbid condition) + 0.84 * 0.10 (baseline utility score was 0.10 greater than 0.50) = 1.434. Taking the inverse logit, this particular patient would have a predicted long-term utility score of: $\frac{e^{1.434}}{1+{e}^{1.434}}$= 0.81

Predicted mean AUC from the regression (with and without imputation) were similar to observed scores from Table 4. For example, a 60 year old male with neuroinvasive disease, one comorbid condition, and a baseline utility score of 0.60 would have a predicted AUC past six months equal to 0.81. Patients with neuroinvasive disease had a mean AUC of 0.80 observed.

## Discussion

We described HRQoL longitudinally as utility scores in a Canada-wide cohort of patients with WNV. At baseline, patients had an impaired HRQoL (mean: 0.59 (95% CI: 0.57, 0.62)) that improved rapidly, and plateaued at about six months of follow-up (mean: 0.77 (95% CI: 0.74, 0.80)). Throughout follow-up, patients with neuroinvasive disease fared consistently worse than their non-neuroinvasive counterparts, although the gap closed by one year. After adjusting for confounding, neuroinvasive disease was no longer a significant predictor of HRQoL. Both the mixed-effects model on HRQoL over time, and the beta regression model on HRQoL post-acute infection showed comorbidities, and baseline HRQoL were significant predictors. This finding is similar to a New York City study that noted better health one year after WNV onset in patients with no comorbidities versus patients with hypertension or diabetes [16]. It reported greater recovery in physical, cognitive, and functional health domains among patients with no comorbidities (risk ratio:2.1 (95% CI: 0.80–5.6)) [16].

Patients after acute infection did not recover to “perfect health”, which would have been indicated by a utility score of 1. It is not known if patients recovered to levels prior to WNV infection since HRQoL data were collected from cohort enrollment onward. Reference utility scores, however, exist among Canadian community-dwellers [17]. The general population without chronic conditions had qualitatively better utility scores than our sample throughout follow-up (mean: 0.93 ± 0.079). The general population in an age group similar to our sample (50 to 59 year olds) had a mean score of 0.92 ± 0.070. These scores were derived from the Health Utilities Index (HUI)-Mark III [18], which uses a time trade-off technique unlike the SF-6D which uses standard gamble. HUI-Mark III also differs in the health domains assessed, notably in vision, hearing, speech, and dexterity.

To our knowledge, this is the first study to report utility scores for the WNV patient population. Such weights are essential in cost-utility analyses for calculating quality-adjusted life-years. Quality-adjusted life-years are a function of health utilities over time. The few existing WNV economic evaluations used utility scores taken from other diseases: unspecified neurologic conditions (0.75) [19]; herpes simplex in the central nervous system, and *Haemophilus influenzae* vaccination sequelae (values not reported) [20]. Keeping in mind future economic evaluations, we present health utilities at different time points, stratified by patient characteristics including neuroinvasive disease, age, sex, and comorbidities, and present summary scores of HRQoL post-acute infection.

The SF-6D instrument incorporates general population weights from the United Kingdom [7], which we applied to patients in Canada where general population weights are unavailable. Valuation of health may differ between the two countries. Other studies examining psychological or physical quality of life domains have used instruments including the Depression Anxiety Stress Scale, Beck Depression Inventory II, Fatigue Severity Scale, Modified Fatigue Impact Scale, Barthel Index of Activities of Daily Living, and Modified Rankin Scale [3, 21–23]. Only Hasbun et al., and Carson et al. used instruments that could generate utilities—namely, SF-36, and its abbreviated version, Short Form 12 (SF-12) [22, 23]. Both the SF-36 and SF-12 are generic rather than disease-specific HRQoL instruments, meaning they can be applied to, and compared across different medical conditions, and patient populations. However, generic instruments may not capture neurological attributes particular to WNV. For instance, fever, stiff neck, headache, weak muscles, gastrointestinal symptoms, disorientation, tremors, convulsions, and paralysis are manifestations of neuroinvasive disease [24]. At this point, WNV-specific HRQoL instruments have not been developed. Neurology-specific instruments may be considered for use in conjunction with general instruments, just as arthritis-specific instruments have been accepted for use in another arbovirus, chikungunya, specifically for its rheumatism sequelae [25].

The two studies that used the Short Form questionnaires may not be comparable to our present work. Hasbun et al. conducted a cross-sectional study on an existing cohort formed in 2002 from Houston, Texas [22]. Patients were originally recruited through local health department surveillance and routine screening of blood donations. The authors assessed 111 patients a median of 6.8 years post-infection (range: 0.1–11 years), and concluded that patients with WNV-associated retinopathy had significantly lower SF-36 scores compared to patients without (mean: 93.2 ± 14.8 versus 100.2 ± 8.3). It is unclear which SF-36 health domain was being reported, and how a mean score was over the maximum of 100. In the second study, Carson et al. recruited 49 patients from North Dakota in 2003 using state-based surveillance, and laboratory records [23]. SF-12 was assessed a mean of 13 months post-diagnosis (range: 10.5–15.8 months). Hospitalized patients had a mean Physical Component Summary score of 43.7 ± 9.3, and Mental Component Summary score of 51.6 ± 11.5. Non-hospitalized patients had statistically similar physical and mental scores (40.8 ± 10.1, and 47.8 ± 10.2, respectively). Comparisons to these two studies are difficult when the present work had different assessment time points, recruitment processes, catchment areas, patient sociodemographics, and numerical HRQoL scales.

There are several limitations to this study. Firstly, there was substantial attrition with 62% of patients lost to follow-up by one year. Specifically, 52% with neuroinvasive disease, and 68% with non-neuroinvasive were lost by then, introducing differential selection bias by oversampling the former subgroup. In the original cohort study, nine patients ended participation after having felt improvements to their health [5]. We may have underestimated HRQoL over the long-term if only the sickest are represented near the end of the study period. However, we may also have overestimated HRQoL due to survival bias where the healthiest patients remain alive through follow-up. We explored multiple imputation of missing data. Re-analysis using imputed datasets showed results similar to the analysis on only the observed data, suggesting robustness. Secondly, long-term HRQoL inferences were limited by a maximum of three years of follow-up. The first human case in Canada was reported in 2002 in Ontario [5]. Continual efforts to collect data can provide insights to neurological, functional, cognitive, and renal sequelae [26–29]. The longest follow-up in a North American cohort was ten years among patients identified through public health surveillance in 2002 in Houston, Texas [27]. While the WNV cohort from which our data originated was the largest in Canada, we only stratified patients by neuroinvasive and non-neuroinvasive diseases. Sample sizes did not allow for further stratification into encephalitis, meningitis, meningoencephalitis, and acute flaccid paralysis. An overall literature gap in syndrome-specific data remains, especially for patients with acute flaccid paralysis and meningoencephalitis [30].

## Conclusions

Given the seasonal epidemics of WNV infections in North America, a greater understanding of HRQoL over the short and long term is warranted. Patients with WNV infection reported low HRQoL during acute illness, but demonstrated rapid improvements by six months of follow-up. Neuroinvasive disease status was not predictive of HRQoL over time. HRQoL accounts for patient preferences, providing important evidence for clinical, and health policy decision-making.

## Additional files

Additional file 1: Table S1. Linear mixed-effects model for predictors of health-related quality of life (measured as untransformed utility scores†) over entire follow-up. (DOCX 15 kb)

Additional file 2: Table S2. Response feature analysis: Utility scores† summarized into mean of long-term utilities. Results are reported as means (95% confidence interval). (DOCX 13 kb)

Additional file 3: Figure S1. Predicted values from best beta-regression model for area under the curve (AUC) past one year for patients with neuroinvasive disease (pink) and non-neuroinvasive disease (blue). (PDF 248 kb)

## Abbreviations

HRQoL: health-related quality of life
HUI: Health Utilities Index
SF-12: Medical Outcomes Survey Short Form 12
SF-36: Medical Outcomes Survey Short Form 36
SF-6D: Medical Outcomes Study Short Form 6D classification
WNV: West Nile virus
