# Supplementary material for: Health-related quality of life in persons with West Nile virus infection: a longitudinal cohort study
Source: Health Qual Life Outcomes. 2017 Oct 23;15:210. doi: 10.1186/s12955-017-0787-5 (PMC5654088; doi:10.1186/s12955-017-0787-5)
Supplement: Supplementary file 1 — Linear mixed-effects model for predictors of health-related quality of life (measured as untransformed utility scores†) over entire follow-up. (DOCX 15 kb) [file 12955_2017_787_MOESM1_ESM.docx]

**ADDITIONAL FILE 1**

**Accompanying the manuscript: “Health-related quality of life in persons with West Nile infection: a longitudinal cohort study”**

# Table S1. Linear mixed-effects model for predictors of health-related quality of life (measured as untransformed utility scores†) over entire follow-up.

|  | **Fixed effects (95% confidence interval)** | | |
| --- | --- | --- | --- |
|  | **Time only model** | **Time + one additional covariate in model** | **All covariates in model** |
| Intercept | 0.71 (0.69, 0.73) | (varies) | 0.71 (0.67, 0.74) |
| Neuroinvasive disease | — | **-0.06 (-0.10, -0.02)** | -0.02 (-0.06, 0.02) |
| Age (per 10 years), centered at 50 years | — | -0.01 (-0.02, 0.01) | 0.0 (-0.02, 0.01) |
| Male | — | **0.04 (0.0, 0.08)** | **0.04 (0.01, 0.07)** |
| Number of comorbid conditions | — | **-0.04 (-0.06, -0.02)** | **-0.04 (-0.05, -0.02)** |
| Baseline utility score, centered at 0.50 | — | **0.40 (0.27, 0.53)** | **0.35 (0.23, 0.47)** |
| Time elapsed since baseline (years) | **0.11 ( 0.09, 0.14)** | (varies) | **0.09 (0.06, 0.12)** |
| Interaction between neuroinvasive disease and years elapsed | — | — | 0.01 (-0.03, 0.05) |

† Utility scores range on a scale from 0 (equivalent to death) to 1 (equivalent to perfect health).

Significant results are **bolded**.

The coefficients represent the change in the mean utility scores when the predictor increases by one unit and the remaining covariates are held constant.
