# Supplementary material for: Health-related quality of life in persons with West Nile virus infection: a longitudinal cohort study
Source: Health Qual Life Outcomes. 2017 Oct 23;15:210. doi: 10.1186/s12955-017-0787-5 (PMC5654088; doi:10.1186/s12955-017-0787-5)
Supplement: Supplementary file 2 — Response feature analysis: Utility scores† summarized into mean of long-term utilities. Results are reported as means (95% confidence interval). (DOCX 13 kb) [file 12955_2017_787_MOESM2_ESM.docx]

**ADDITIONAL FILE 2**

**Accompanying the manuscript: “Health-related quality of life in persons with West Nile infection: a longitudinal cohort study”**

# Table S2. Response feature analysis: Utility scores† summarized into mean of long-term utilities. Results are reported as means (95% confidence interval).

|  | Mean of long-term utilities ^a^ | |
| --- | --- | --- |
|  | **Six months onward** | **One year onward** |
| Total cohort | 0.78  (0.75, 0.80)  n= 108 | 0.81  (0.78, 0.84)  n= 59 |
| Neuroinvasive | 0.75  (0.71, 0.79)  n= 45 | 0.80  (0.76, 0.85)  n= 30 |
| Nonneuroinvasive | 0.80  (0.77, 0.83)  n= 56 | 0.81  (0.76, 0.86)  n= 29 |

† Utility scores range on a scale from 0 (equivalent to being dead) to 1 (equivalent to perfect health). Larger values indicate better health-related quality of life. Scores are derived from the Medical Outcomes Study Short-Form-6D health state classification.

* One patient missing utility scores at all visits

^a^ Long-term score is the patient’s mean score of all study visits either (i) six months and onward, or (ii) one year and onward
