# Supplementary material for: Health-related quality of life in persons with West Nile virus infection: a longitudinal cohort study
Source: Health Qual Life Outcomes. 2017 Oct 23;15:210. doi: 10.1186/s12955-017-0787-5 (PMC5654088; doi:10.1186/s12955-017-0787-5)

ADDITIONAL FILE 3

Accompanying the manuscript: "Health-related quality of life in persons with West Nile infection: a longitudinal cohort study"

Figure S1. Predicted values from best beta-regression model for area under the curve (AUC) past one year for patients with neuroinvasive disease (pink) and nonneuroinvasive disease (blue).

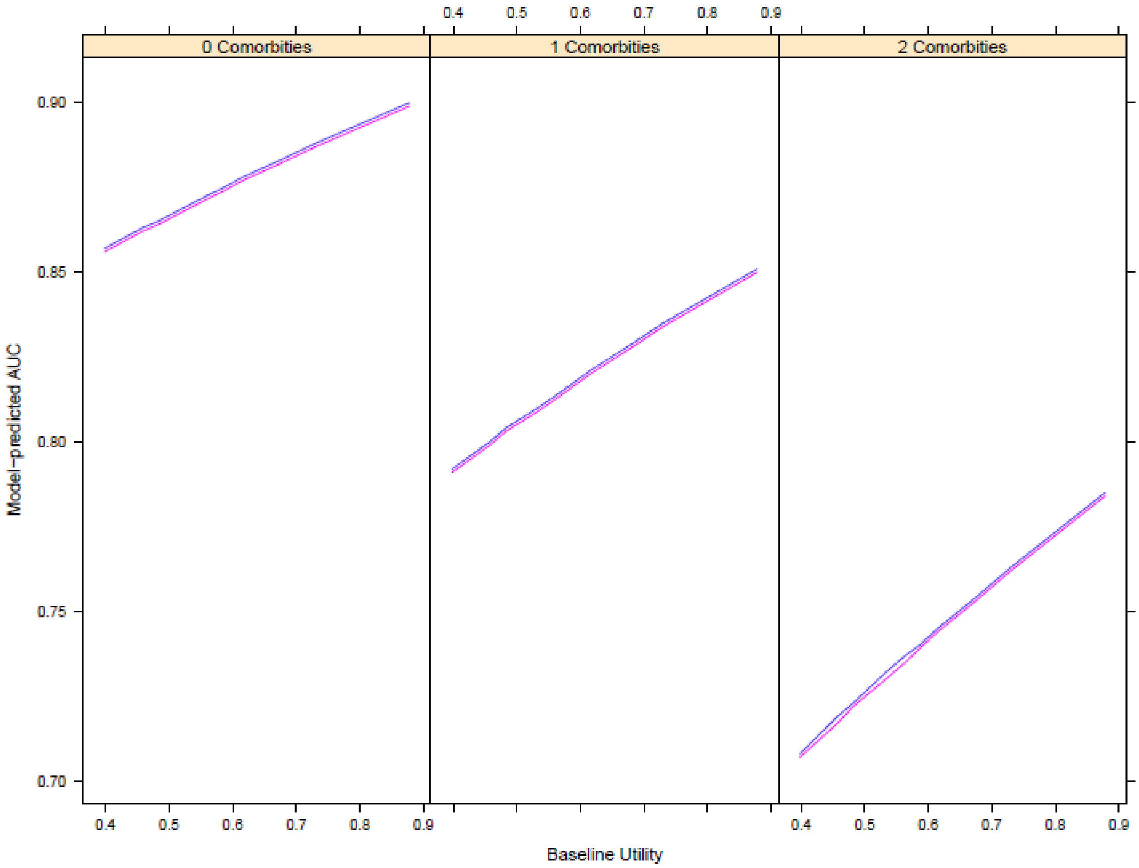

Supplement: Supplementary file 3 — Predicted values from best beta-regression model for area under the curve (AUC) past one year for patients with neuroinvasive disease (pink) and non-neuroinvasive disease (blue). (PDF 248 kb) [file 12955_2017_787_MOESM3_ESM.pdf]
